# Distinctive Subcellular Inhibition of Cytokine-Induced Src by Salubrinal and Fluid Flow

**DOI:** 10.1371/journal.pone.0105699

**Published:** 2014-08-26

**Authors:** Qiaoqiao Wan, Wenxiao Xu, Jing-long Yan, Hiroki Yokota, Sungsoo Na

**Affiliations:** 1 Department of Biomedical Engineering, Indiana University-Purdue University Indianapolis, Indianapolis, Indiana, United States of America; 2 Department of Orthopedics, Second Clinical Hospital of Harbin Medical University, Harbin, China; 3 Department of Anatomy and Cell Biology, Indiana University School of Medicine, Indianapolis, Indiana, United States of America; University of Sydney, Australia

## Abstract

A non-receptor protein kinase Src plays a crucial role in fundamental cell functions such as proliferation, migration, and differentiation. While inhibition of Src is reported to contribute to chondrocyte homeostasis, its regulation at a subcellular level by chemical inhibitors and mechanical stimulation has not been fully understood. In response to inflammatory cytokines and stress to the endoplasmic reticulum (ER) that increase proteolytic activities in chondrocytes, we addressed two questions: Do cytokines such as interleukin 1 beta (IL1β) and tumor necrosis factor alpha (TNFα) induce location-dependent Src activation? Can cytokine-induced Src activation be suppressed by chemically alleviating ER stress or by applying fluid flow? Using live cell imaging with two Src biosensors (i.e., cytosolic, and plasma membrane-bound biosensors) for a fluorescence resonance energy transfer (FRET) technique, we determined cytosolic Src activity as well as membrane-bound Src activity in C28/I2 human chondrocytes. In response to TNFα and IL1β, both cytosolic and plasma membrane-bound Src proteins were activated, but activation in the cytosol occurred earlier than that in the plasma membrane. Treatment with salubrinal or guanabenz, two chemical agents that attenuate ER stress, significantly decreased cytokine-induced Src activities in the cytosol, but not in the plasma membrane. In contrast, fluid flow reduced Src activities in the plasma membrane, but not in the cytosol. Collectively, the results demonstrate that Src activity is differentially regulated by salubrinal/guanabenz and fluid flow in the cytosol and plasma membrane.

## Introduction

Chondrocytes are a predominant cell type present in articular cartilage, whose integrity is jeopardized in joint degenerative diseases such as osteoarthritis (OA) [1]. In the chondrocytes [2]–[4] as well as the synovial tissues [5], [6] of patients with OA, the elevated level of inflammatory cytokines such as interleukin 1β (IL1β) and tumor necrosis factor α (TNFα) have been reported. These cytokines contribute to degrading cartilage matrix by increasing activities of proteolytic enzymes, including matrix metalloproteinases (MMPs) and ADAMTS (a disintegrin and metalloproteinase with thrombospondin motifs) [7]. In addition to their contribution to proteolytic enzymes, these cytokines adversely affect anabolic activity of chondrocytes by inhibiting the production of proteoglycans and type II collagen [8], [9]. Therefore, blocking the action of these cytokines is a potential strategy to prevent cartilage degradation.

The articular cartilage is primarily subjected to compression, which results in complex mechanical stimuli including tissue deformation, fluid flow-induced shear stress, and hydrostatic pressure [10]. It has been reported that moderate, physiological mechanical loading contributes to maintenance of cartilage homeostasis (reviewed in [11], [12]). Application of gentle loading, for example, is shown to inhibit IL1-induced matrix degradation [13] as well as expression of MMPs and ADAMTS [14], [15]. Importantly, integrin-dependent signaling is linked to IL1-induced signaling in chondrocytes [16]–[19]. Src is one of the integrin-dependent signaling proteins involved in mechanotransduction [20], and it plays critical roles in various cellular processes including proliferation, apoptosis, migration, adhesion, and differentiation [21]. To mediate such a variety of cellular processes, Src's distinct subcellular activation pattern is required. Src is mainly stationed in the cytosol near the endosomes [22], and activation of Src requires its translocation to the plasma membrane [23] through the cytoskeleton [24]. While Src is known to regulate proliferation and differentiation of chondrocytes [25], its responses to inflammatory cytokines and fluid flow, particularly at the subcellular level, have not been well understood.

In addition to cytokines and mechanical loading, cellular stress to the endoplasmic reticulum (ER) is known to affect chondrocyte functions [26] and expression of MMPs [27]. We have previously reported that salubrinal, a chemical agent that reduces ER stress [28], inhibits IL1β- and TNFα-induced MMP activities by inhibiting dephosphorylation of eukaryotic translation initiation factor 2 alpha (eIF2α) [29]. Phosphorylation of eIF2α plays a crucial role in regulating pro-survival cellular pathways. In response to various environmental stresses including ER stress, viral infection, and oxidative stress, eIF2α is phosphorylated for reducing global translation, allowing cells to activate a group of genes important for survival [30]. Although phosphorylation of eIF2α inhibits integrin-mediated signaling and subsequently reduce MMP activities in chondrocytes [29], little has been known about the role of eIF2α in Src regulation.

In the present study, we investigated the role of eIF2α and mechanical force in Src activity in chondrocytes. In order to visualize subcellular activity pattern of Src in chondrocytes, we used live cell imaging in conjunction with two types of fluorescence resonance energy transfer (FRET)-based Src biosensors that selectively target lipid rafts of the plasma membrane and cytosol. IL1β and TNFα were used as inflammatory cytokines. Three levels of fluid flow were applied to the cells, and the role of mechanical stimulation in Src activity was examined. Salubrinal and guanabenz, the other agent that inhibits dephosphorylation of eIF2α, as well as siRNA specific to eIF2α were used to investigate the role of eIF2α in Src activity.

## Materials and Methods

### Src biosensors

**Figure 1:**
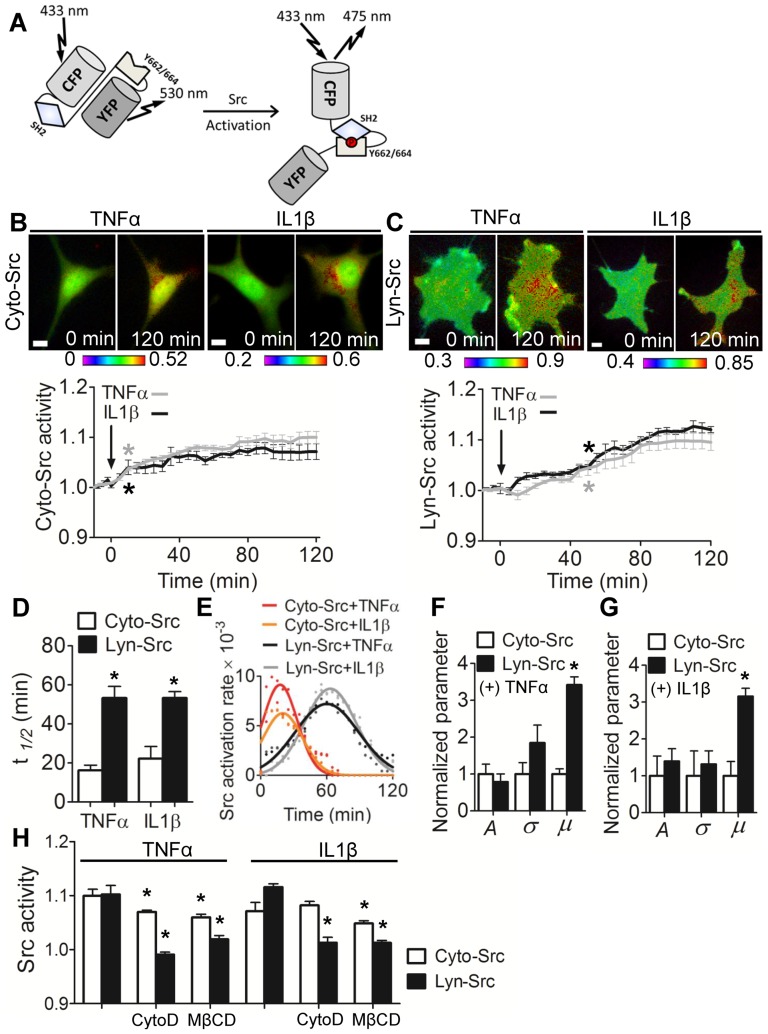
Differential dynamics of Cyto-Src and Lyn-Src activation by TNFα and IL1β. (A) A schematic illustration of the structure and activation mechanism of the FRET-based Src biosensor. (B, C) The FRET ratio images and time courses of Cyo-Src activities (B) and Lyn-Src activities (C) under treatment with TNFα (gray) and IL1β (black). Color bars represent emission ratio of CFP/YFP of the biosensor, an index of Cyto-Src activation. The FRET ratio images were scaled according to the corresponding color bar. For each time-lapse imaging experiment, the images from the same cell were taken. The CFP/YFP emission ratios were averaged over the whole cell and were normalized to time 0. *n* = 8 (TNFα), 9 (IL1β) cells in (B); 6 (TNFα), 9 (IL1β) in (C). Scale bars, 10 µm. (D) The t_1/2_ values of Src response to TNFα and IL1β. * *p*<0.05 between Cyto-Src and Lyn-Src. (E) Gaussian function curves determined by curve fitting of the rate of mean FRET changes over time under cytokine treatment. (F, G) The normalized values of *A*, µ, and *σ* for Cyto-Src and Lyn-Src activities that were calculated by parameter fitting in cells under treatment with TNFα (F) or IL1β (G). *n*>6 cells. * *p*<0.05 between Cyto-Src and Lyn-Src. (H) The response of Src activities to cytokines in cells pretreated with CytoD (1 µg/ml, 1 h) to disrupt actin filaments or MβCD (10 mM, 1 h) to extract cholesterol from the plasma membrane. The Src activities at 2 hours after cytokine treatment were normalized to time 0. *n*>9 cells. * *p*<0.05 compared to the group treated with a corresponding cytokine alone.

FRET-based, cyan fluorescent protein (CFP)-yellow fluorescent protein (YFP) biosensors were used for monitoring Src activities in the cytosol (Cyto-Src) and lipid rafts of the plasma membrane (Lyn-Src) [31], [32]. The Cyto-Src biosensor consists of CFP, a binding domain of an effector protein (SH2 domain), a truncated Src substrate peptide and YFP (Fig. 1A). The lipid rafts-targeted Src biosensor was produced by fusing acylation substrate sequences derived from Lyn kinase to the N-terminal of Cyto-Src biosensor [31]. Src activation promotes the intramolecular binding of the SH2 domain to the truncated Src domain within the biosensor, which leads to a conformational change of the biosensor and the decrease of FRET efficiency from CFP to YFP (Fig. 1A). Hence, Src activity can be visualized as changes of the emission ratio of the CFP/YFP. The specificity of the biosensors has been well characterized previously [31], [32].

### Chemical reagents and eIF2α siRNA

Two types of proinflammatory cytokines, TNFα (Sigma; 10 ng/ml) and IL1β (Sigma; 1 ng/ml), were used. Salubrinal and guanabenz (both from Tocris Bioscience), inhibitors of eIF2α phosphatase, were used to test the effect of phosphorylation of eIF2α on Src activity [28], [33]. We also used eIF2α siRNA and non-specific control (NC) siRNA (Origene). Cytochalasin D (Sigma; 1 µg/ml) was used to disrupt actin filaments. Methyl-beta-cyclodextrin (MβCD; Sigma; 10 mM) was used to extract cholesterol from the lipid rafts of the plasma membrane.

### Cell culture, transfection, and Western blotting

The human chondrocyte cell line C28/I2 was used [34]. Cells were cultured in Dulbecco's modified Eagle's medium (DMEM; Lonza) containing 10% FBS (Hyclone) and 1% penicillin/streptomycin (Lonza), and maintained at 37°C and 5% CO_2_ in a humidified incubator. Neon transfection system (Invitrogen) was used to transfect Src biosensors into the cells. After transfection, the cells were transferred to a type I collagen-coated glass bottom dish or µ-slide cell culture chamber (Ibidi) and incubated in DMEM containing 0.5% FBS for 24–36 h before imaging experiments. For Western blotting, cells were grown in the presence and absence of salubrinal or guanabenz and lysed in a radioimmunoprecipitation assay (RIPA) buffer. Isolated proteins were fractionated using 10% SDS gels and electro-transferred to Immobilon-P membranes. The membrane was incubated for 1 h with primary antibodies followed by 45 min incubation with secondary antibodies conjugated with horseradish peroxidase (Cell Signaling). We used antibodies against eIF2α (Cell Signaling), phosphorylated eIF2α (p-eIF2α; Thermo), and β-actin (Sigma). Signal intensities were quantified with a luminescent image analyzer (LAS-3000, Fujifilm).

### Shear stress application

Fluid flow-induced shear stress has been shown to play a crucial role in the development and progression of osteoarthritis [35]. During imaging, a unidirectional flow was applied to the cells grown in the µ-slide cell culture chamber (Ibidi) at 37°C [36], [37]. The chamber was perfused with HEPES-buffered (20 mM), phenol red-free DMEM without serum to maintain the pH at 7.4. Because the shear stress of 2–10 dynes/cm^2^ has been shown to affect chondrocyte signaling and metabolism either positively or negatively [37]–[40], we used this flow range in this study. The shear stress was applied to the cells by controlling the flow rate of a peristaltic pump (Cole-Parmer). A pulse dampener (Cole-Parmer) was used to minimize pulsation of the flow due to the pump.

### Microscopy and image analysis

Images were obtained by using a Nikon Ti-E inverted microscope equipped with an electron-multiplying charge-coupled device (EMCCD) camera (Evolve 512; Photometrics), a filter wheel controller (Sutter Instruments), and a Perfect Focus System (Nikon) that maintains the focus during time-lapse imaging. The following filter sets (Semrock) were used: CFP excitation: 438/24 (center wavelength/bandwidth in nm); CFP emission (483/32); YFP (FRET) emission: 542/27. To minimize photobleaching, cells were illuminated with a 100 W Hg lamp through an ND64 (∼1.5% transmittance) neutral density filter. Time-lapse images were acquired at intervals of 2 min with a 40× (0.75 numerical aperture) objective. FRET images for Src activity were generated with NIS-Elements software (Nikon) by computing an emission ratio of CFP/YFP for individual cells over time. The FRET ratio images were scaled according to the color bar. To quantify the kinetics of the FRET responses of Src biosensors, the discrete time derivatives of the emission ratios, *Y*, were calculated and the associated curves were fitted using the Gaussian functions: *Y*  =  *A*•exp[-0.5•((*t*-µ)/*σ*)^2^]. The parameter *A* represents the maximal rate of FRET ratio change upon stimulation, µ the time point where the rate of FRET ratio change reaches the maximal value, and *σ* the duration of the rate of FRET ratio change. The rate of FRET ratio change in response to cytokines was assumed to follow a Gaussian distribution. This assumption was tested by the D'Agostino-Pearson (omnibus K2) normality test [41].

### Statistical analysis

Statistical data are presented as the mean ± standard error of the mean (SEM). One-way ANOVA followed by Dunnett's post hoc test was used to determine the statistical differences. Student's *t*-test was used to compare two groups. Statistical analyses were conducted using Prism 5 software (GraphPad Software). *p*<0.05 was considered significant. In the time course data, * indicates the time point after which the Src activity becomes significantly different from that at 0 min.

## Results

### Activation of Src at different subcellular locations by TNFα and IL1β

Since Src can be activated at different subcellular locations [42], we first tested whether Cyto-Src in the cytosol and Lyn-Src in the lipid rafts of the plasma membrane would be differently regulated by TNFα and IL1β. In C28/I2 human chondrocytes, Cyto-Src activities were increased by treatment of TNFα or IL1β and reached a maximal at 90 min by TNFα (10.0%) and at 80 min by IL1β (7.1%) (Fig. 1B). Its activities after 10 min of the cytokine treatment were significantly different from those at time 0 (*p*<0.05), and the activation level was maintained for 2 h. Lyn-Src activities were also increased by TNFα or IL1β, while they showed a slower increase than that of Cyto-Src (Fig. 1C). The FRET ratio increased and reached the peak value at 120 min by TNFα (10.2%) and at 110 min by IL1β (11.6%). The Lyn-Src activities after 50 min of the cytokine treatment were significantly different from those at time 0 (*p*<0.05). We also observed that cytokine-induced Lyn-Src activities were initially decreased (Fig. 1C). However, the decreased Src activity following cytokine treatment was not significantly different from that at 0 min.

Since the temporal profiles of Cyto-Src and Lyn-Src activities were different, we determined t_½_, which measured the time required for Src to reach the half-maximal activity level (Fig. 1D). The result showed that the mean and standard deviation of t_½_ of Cyto-Src (TNFα: 16.25±2.63 min; and IL1β: 22.22±6.24 min) were significantly lower than those of Lyn-Src (TNFα: 53.33±5.87 min; and IL1β: 53.33±3.23 min). To evaluate a rate of activation changes in response to TNFα and IL1β, we computed time derivatives of activation levels and the estimated rate was fitted to a Gaussian distribution function (Fig. 1E). Based on the D'Agostino-Pearson (omnibus K2) normality test, the *p* values for all experimental conditions were higher than the cut-off value, 0.05, indicating that the data follow a Gaussian distribution. In the fitting, the rate of the changes in Cyto-Src reached the maximal earlier than that in Lyn-Src (Fig. 1E), consistent with t_½_ data (Fig. 1D). Compared to Lyn-Src, Cyto-Src activities showed significantly lower mean time parameters in the Gaussian curves (Cyto-Src+TNFα: 17.59±2.51; Cyto-Src+IL1β: 20.02±7.78; Lyn-Src+TNFα: 60.11±3.87; and Lyn-Src+IL1β: 63.19±4.37). However, differences in the level and duration of Src activation were not statistically significant (Fig. 1F–G).

### Effects of actin cytoskeleton and lipid rafts on cytokine-induced Lyn-Src activation

Since activation of Src is associated with its translocation to the plasma membrane via the actin cytoskeleton [43], we examined the roles of the actin cytoskeleton and lipid rafts in the responses to cytokines. Cells were pretreated with Cytochalasin D (CytoD) for 1 h to disrupt the actin cytoskeleton or with MβCD for 1 h to extract cholesterol from the plasma membrane. CytoD partially blocked Cyto-Src activation, and it completely inhibited Lyn-Src activation (Fig. 1H). MβCD reduced both Cyto-Src and Lyn-Src activations, although to a lesser degree to Cyto-Src (Fig. 1H). Collectively, these data suggest that the actin cytoskeleton and lipid rafts are essential components for cytokine-induced Lyn-Src activation (Fig. 1D–H).

### Inhibition of Cyto-Src by salubrinal and guanabenz

**Figure 2:**
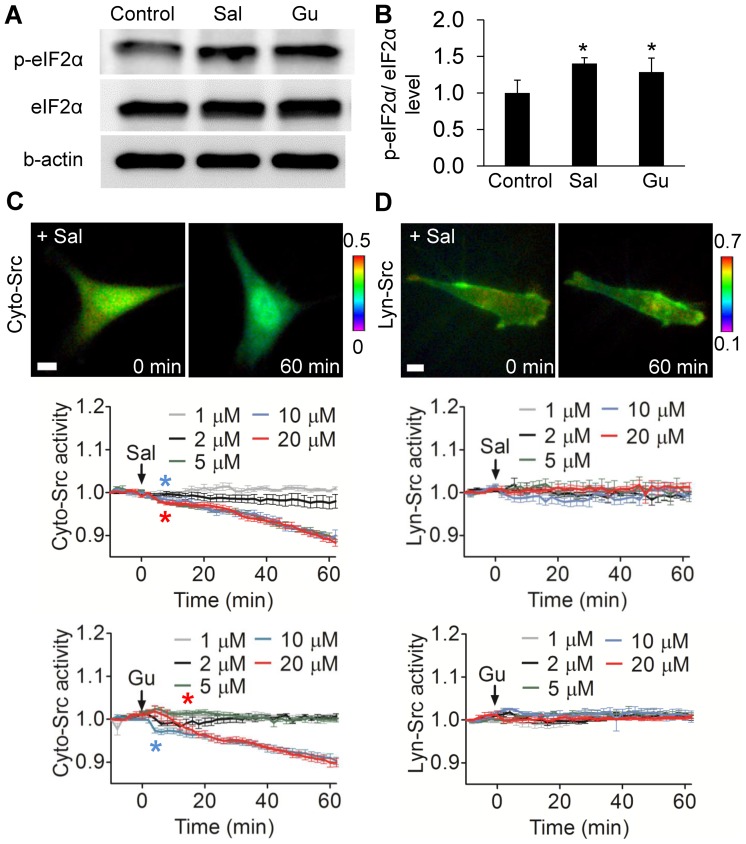
Salubrinal (Sal) and guanabenze (Gu) increase phosphorylation of eIF2α and decrease Cyto-Src activities. (A) Western blots showing the elevated level of p-eIF2α by salubrinal and guanabenz. (B) Staining intensity of p-eIF2α, normalized by intensity of eIF2α. * *p*<0.05. (C) Cyto-Src activity by salubrinal and guanabenz. (D) Lyn-Src activity by salubrinal and guanabenz. Scale bars, 10 µm. *n*>7 cells.

Western blotting revealed that incubation with salubrinal and guanabenz elevated the phosphorylation level of eIF2α by 40±9% (*p*<0.01) and 29±19% (*p*<0.05), respectively (Fig. 2A–B). Salubrinal decreased Cyto-Src activity in a dose-dependent manner (Fig. 2C). Although a lower concentration (1 and 2 µM) did not detectably alter Cyto-Src activity, salubrinal at 5, 10, and 20 µM significantly decreased it (5 µM: 10.9%; 10 µM: 10.0%; and 20 µM: 11.7% after 60 min). Cyto-Src activities after 8 min were significantly different from those at 0 min (*p*<0.001). Guanabenz at 10 and 20 µM also decreased Cyto-Src activity, while lower concentrations did not affect it (10 µM: 9.7%; 20 µM: 10.1% at 60 min). Its activities after guanabenz treatment (4 min at 10 µM; 14 min at 20 µM) were significantly different from those at time 0 (*p*<0.001). In contrast to Cyto-Src, Lyn-Src activity was not altered either by salubrinal or guanabenz (Fig. 2D and Fig. S1).

**Figure 3:**
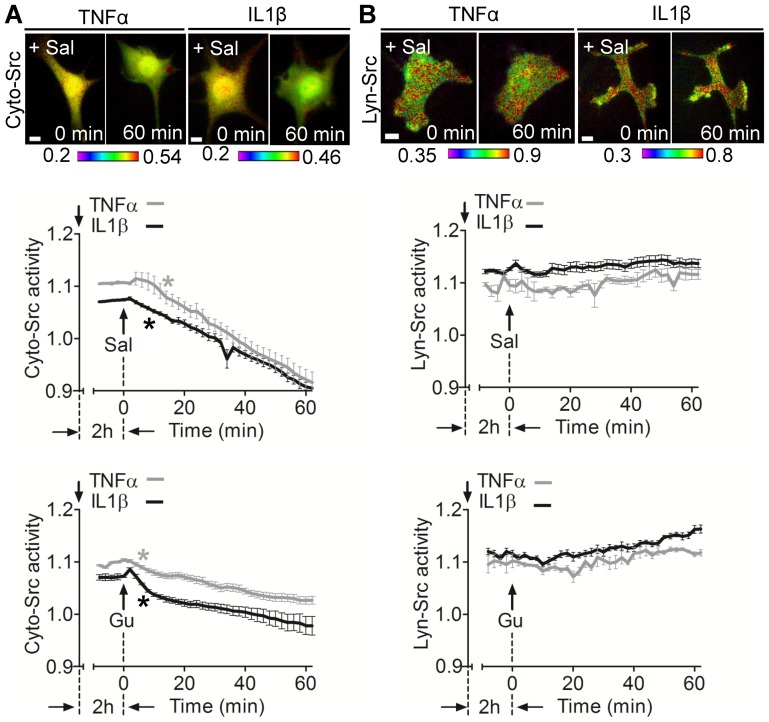
Salubrinal and guanabenz inhibit cytokine-induced Cyto-Src activity. (A, B) C28/I2 cells transfected with either Cyto-Src or Lyn-Src biosensor were pretreated with TNFα or IL1β for 2 hours before incubating with salubrinal or guanabenz. (A) Effect of Salubrinal and guanabenz on cytokine-induced Cyto-Src activity. (B) Cytokine-induced Lyn-Src activity is not altered by salubrinal or guanabenz. Scale bars, 10 µm. *n*>7 cells.

In response to TNFα or IL1β, both salubrinal and guanabenz significantly reduced cytokine-induced Cyto-Src activities (Fig. 3A and Fig. S2), but they did not inhibit cytokine-induced Lyn-Src activation (Fig. 3B).

### Involvement of eIF2α in the inhibitory effect of salubrinal on Cyto-Src

**Figure 4:**
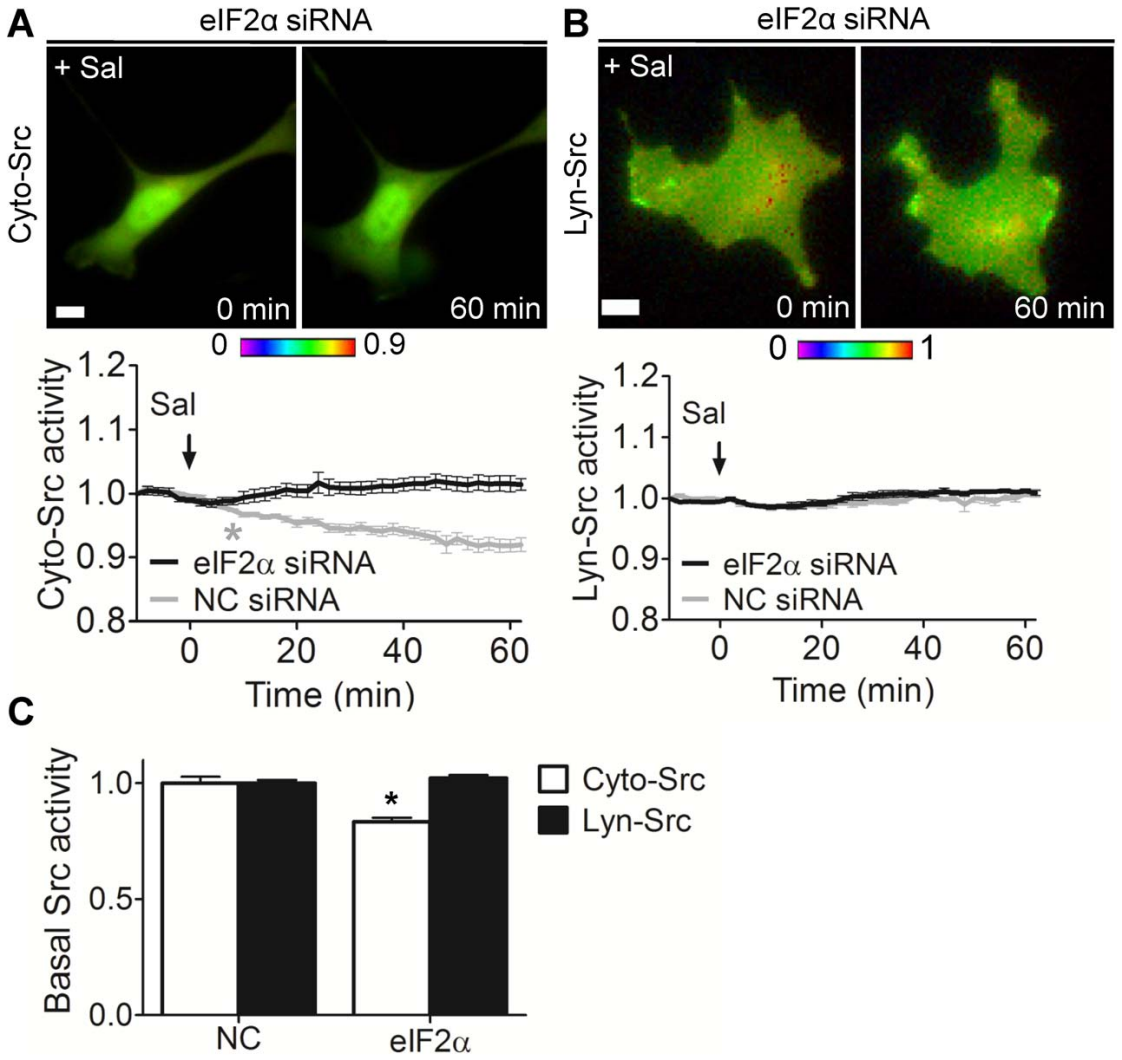
Involvement of eIF2α in salubrinal-driven Cyto-Src activity. (A, B) C28/I2 cells were cotransfected with Cyto-Src or Lyn-Src biosensor, and eIF2α or NC siRNA, and then treated with 10 µM salubrinal for 1 hour during imaging. (A) eIF2α siRNA blocks inhibitory effect of salubrinal on Cyto-Src activity. (B) Lyn-Src activity is not altered by eIF2α siRNA. Scale bars, 10 µm. *n*>7 cells. (C) The basal level of Src activity in C28/I2 cells expressing NC or eIF2α siRNA. *n*>7 cells. * *p*<0.05 compared to the corresponding NC group.

Salubrinal and guanabenz is known to increase the phosphorylation level of eIF2α (Fig. 2A, B) [28], [33]. To examine whether the inhibition of Src by salubrinal is associated with eIF2α, we employed eIF2α siRNA. The partial silencing of eIF2α abolished the inhibitory effect of salubrinal on Cyto-Src as compared to the cells treated with non-specific control siRNA (Fig. 4A). However, salubrinal did not alter Lyn-Src activity by eIF2α siRNA (Fig. 4B). Silencing eIF2α significantly decreased the basal level of Cyto-Src activity, but not that of Lyn-Src activity as compared to the NC siRNA-treated Src activity (Fig. 4C).

### Magnitude-dependent regulation of Lyn-Src by fluid flow

**Figure 5:**
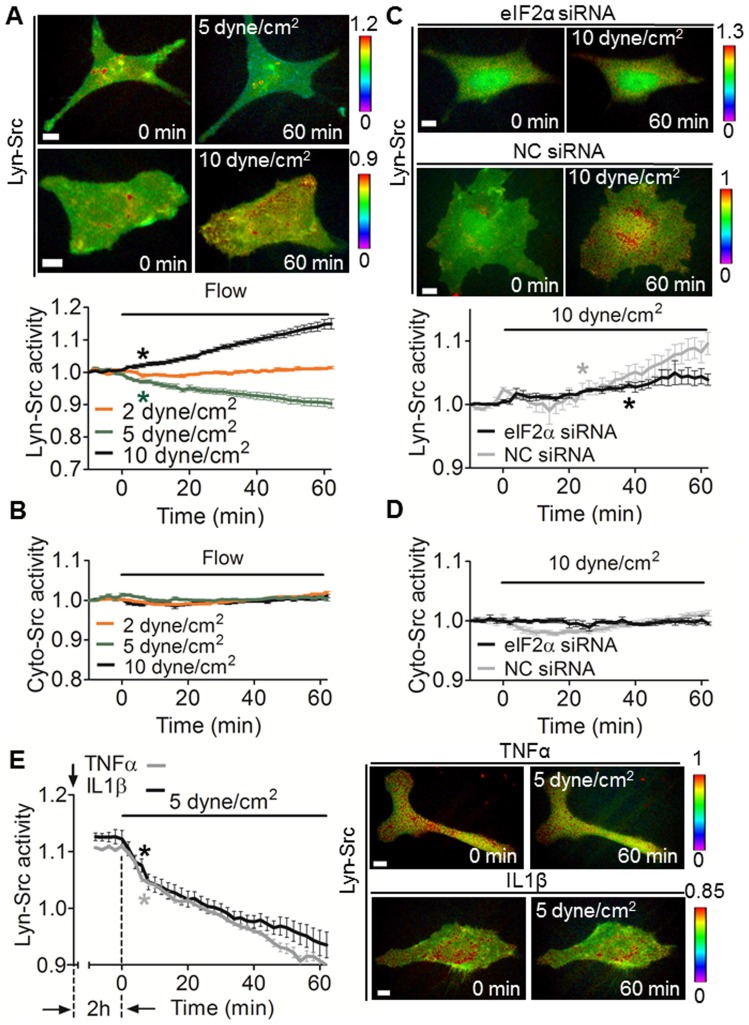
Fluid flow magnitude-dependent Lyn-Src activity. (A) Selective Lyn-Src activities in response to different magnitudes of fluid flow. *n*>7 cells. (B) Cyto-Src activity is not altered by fluid flow. *n*>7 cells. (C, D) C28/I2 cells were cotransfected with either Lyn-Src or Cyto-Src biosensor and eIF2α siRNA or NC siRNA, and then subjected to fluid flow (10 dynes/cm^2^) during FRET imaging. (C) Lyn-Src activity under fluid flow. *n*>7 cells. (D) Cyto-Src activity under fluid flow. *n*>7 cells. (E) Lyn-Src activity in cytokine-treated cells under fluid flow(5 dynes/cm^2^). Cells transfected with a Lyn-Src biosensor were pretreated with cytokines for 2 hour before FRET imaging. *n*>7 cells. Scale bars, 10 µm.

To determine whether mechanical stimulation affects Src activities, we applied uniform fluid flow with shear stress at 2, 5, or 10 dynes/cm^2^ for 1 h. Lyn-Src was responsive to fluid flow in a magnitude-dependent manner (Fig. 5A). In response to shear stress at 5 dynes/cm^2^, a rapid inhibition of Lyn-Src activity was observed (9.7% decrease). In contrast, shear stress at 10 dynes/cm^2^ led to its activation (14.9% increase). However, Cyto-Src activity was not altered at any magnitude of shear stress (Fig. 5B).

Silencing eIF2α significantly reduced fluid flow-induced Lyn-Src activation, although eIF2α siRNA did not completely abolish the activation (3.9% increase at 60 min, Fig. 5C). Note that transfection of NC siRNA led to a significant Lyn-Src activation by fluid flow (9.6% increase at 60 min). Unlike Lyn-Src, Cyto-Src in C28/I2 cells transfected with eIF2α or NC siRNA did not respond to fluid flow (Fig. 5D). These results demonstrate that eIF2α is at least in part involved in the Lyn-Src response to fluid flow. To further examine the observed inhibitory effect of fluid flow at 5 dynes/cm^2^ on Lyn-Src activities, we pretreated cells with TNFα or IL1β for 2 h before the application of fluid flow. Shear stress at 5 dynes/cm^2^ substantially reduced the activation level of cytokine-induced Lyn-Src (TNFα: 10.1% decrease at 60 min; and IL1β: 6.5% decrease at 60 min) (Fig. 5E).

## Discussion

In this study we employed live cell imaging in conjunction with FRET-based Src biosensors to determine the spatiotemporal activities of Src in C28/I2 human chondrocytes. We used two types of the Src biosensors specific in the cytosol and lipid rafts of the plasma membrane. We first demonstrated that Src proteins in the cytosol and lipid rafts were activated by TNFα and IL1β with distinct dynamic patterns. Although the role of the inflammatory cytokines in regulating Src activation has been documented [44], its spatiotemporal activation pattern has not been known. We observed that the cytokine-induced Src activation occurred earlier in the cytosol than that in the lipid raft region of the plasma membrane. Since it is considered that Src activation requires its translocation to the plasma membrane via the actin cytoskeleton [43], our observations suggest that upon stimulation, Src moves quicker to the non-lipid rafts than to the lipid rafts of the plasma membrane. Taken together, the result is consistent with previous studies showing that translocation of Src from the cytosol to the non-raft region is faster than that to the raft region of the plasma membrane [45], [46].

It has been reported that TNFα and IL1β differently affect degenerative joint diseases such as osteoarthritis [47], [48]. However, their differential effects on Src activity are not known. We observed that Cyto-Src activities by TNFα were significantly higher than those by IL1β, while Lyn-Src activities under the two cytokines were not significantly different. Although the exact mechanism is not clear, it is possible that Cyto-Src and Lyn-Src would differently interact with inflammatory signaling components, such as TNF receptor DEATH domain (TRADD) [49], TRAF2 [50], and GRB2 [51]. Another possibility is that TNFα would stimulate the release of IL1β [52], which may further increase TNFα-induced Cyto-Src activity. In this study, however, the primary aim was to evaluate the role of mechanical force and salubrinal in the cytokine-induced Src. Investigating Src responses to different cytokines or interaction between TNFα and IL1β is an important subject. Further consideration is necessary to elucidate the molecular mechanism underlying the differential Src activities at different subcellular locations in response to various cytokines.

**Figure 6:**
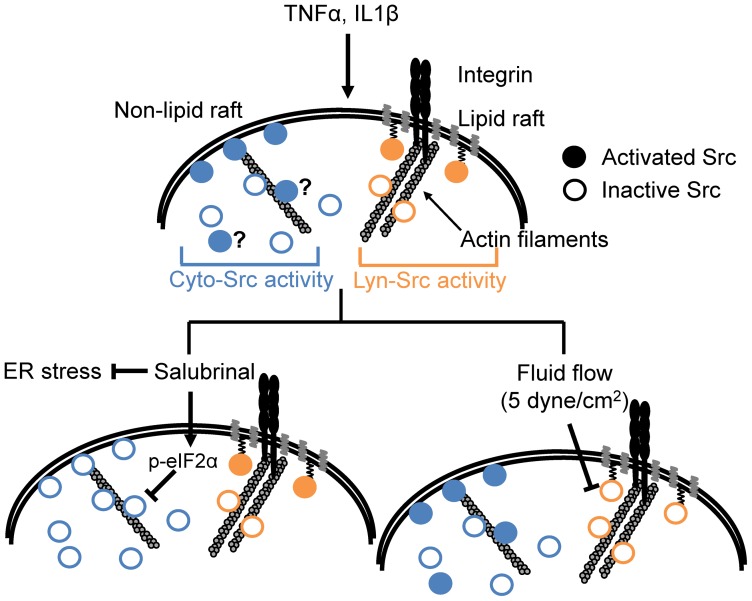
A proposed model of distinctive Src activities at different subcellular locations. TNFα and IL1β activate Src kinases at the cytoplasm and lipid rafts of the plasma membrane, and actin cytoskeleton and lipid rafts are essential components of the Lyn-Src activation. Salubrinal can inhibit Src kinases in the cytoplasm through phosphorylation of eIF2α, but not in the lipid rafts of the plasma membrane. In contrast, fluid flow at 5 dynes/cm^2^ decreases integrin-mediated Src kinases in the lipid rafts of the plasma membrane, but it did not significantly affect the level of Src activation in the cytoplasm.

There are several lines of evidence that Src can be activated at the different locations within the cell, such as in the cytoplasm, along the actin cytoskeleton, or on the plasma membrane [24], [32], [43], [53]. Thus, we evaluated the possibility that Src may be activated in the cytoplasm before it is mobilized to the cell periphery or plasma membrane upon cytokine stimulation. To test this possibility, we disrupted the actin cytoskeleton and monitored Src activity in the cytosol and lipid rafts of the plasma membrane. While disruption of the actin cytoskeleton using cytochalasin D blocked Src activation in the lipid rafts, it did not completely inhibit cytosolic Src activation, suggesting that some population of cytosolic Src may be activated in the cytoplasm, without translocation through the actin cytoskeleton (Fig. 6). These results support the previous findings that activation of Src at the lipid rafts of the plasma membrane requires its translocation through the actin cytoskeleton [53] and that Src can be activated in the cytoplasm [24], [32]. However, our data cannot distinguish whether cytosolic Src near the plasma membrane outside the lipid rafts, which may not require translocation, is activated or other focal adhesion proteins at the plasma membrane, such as vinculin [54] or talin [55], are involved in this cytosolic Src activation. Further studies are needed to elucidate the underlying mechanism for this dynamically distinct Src activation at different cellular compartments.

We have previously reported that the inhibition of ER stress through eIF2α phosphorylation can decrease expression and activity of degrading enzymes such as MMP13 in C28/I2 human chondrocytes [29]. Here, we used two inhibitors for eIF2α dephosphorylation, salubrinal and guanabenz, and tested whether alleviating ER stress by eIF2α phosphorylation could attenuate the inflammatory cytokine-induced Src activation. We observed that the cytokine-induced Src in the cytosol, but not in the lipid rafts of the plasma membrane, was inhibited by salubrinal or guanabenz. This result suggests that Src at different compartments within the cell may be regulated by different mechanisms; cytosolic Src is downregulated by eIF2α phosphorylation, but Src in the lipid rafts may not be a critical signaling node within an ER stress signaling pathway (Fig. 6).

Our results further suggest that these distinct responses of Src activities are differently regulated by fluid flow. In contrast to the nonresponsive Src activity in the lipid rafts of the plasma membrane by salubrinal or guanabenz, fluid flow-induced shear stress (5 dynes/cm^2^) was able to significantly inhibit the cytokine-induced Src activity at the lipid rafts. This result is consistent with previous findings that lipid rafts of the plasma membrane are involved in Src mechanotransduction [31], [56]. On the contrary, cytosolic Src was not responsive to the shear stress. We previously reported that local mechanical force applied from the cell surface using a small (4.5 µm in diameter) magnetic bead induces highly localized cytosolic Src activity and does not yield global FRET changes of the Src biosensors within the cytoplasm [32]. We do not know whether cytosolic Src does not respond to shear stress that is evenly distributed over the cell surface, or activation of the cytosolic Src by shear stress is not sufficient to yield detectable FRET changes from a substantial pool of Src biosensors in the cytosol [31]. We also observed that Src activities at the lipid rafts of the plasma membrane are selectively up- or down-regulated by different magnitudes of shear stress; moderate (5 dynes/cm^2^) and high (10 dynes/cm^2^) shear stress decrease and increase Src activity in the lipid rafts, respectively. This result is consistent with previous reports on fluid flow magnitude-dependent small GTPase RhoA activities [37] and MMP13 activities [57] in chondrocytes.

In summary, our findings of the distinct activation patterns of Src kinases suggest the critical role of mechanical loading and inhibition of ER stress through phosphorylation of eIF2α in arthritic cartilage (Fig. 6). By selectively regulating subcellular Src kinases, fluid flow and a chemical agent that inhibits ER stress appear to interact with Src-dependent regulatory pathways important for chondrogenesis and cartilage maintenance [25], [58]. The work herein suggests that a proper combination of chemical and mechanical stimuli may present a potential therapeutic strategy for prevention of cartilage loss in joint diseases such as OA. Further study on the signaling pathways connecting subcellular Src kinases to proteolytic gene expression in chondrocytes may benefit in developing a therapeutic strategy for joint diseases.

## Supporting Information

Figure S1 **Lyn-Src response to salubrinal (10 µM).** Two hour-imaging data shows that salubrinal does not affect Lyn-Src activity. *n* = 7 cells.
(TIFF)

Figure S2 **Cyto-Src activity of a representative cell in response to to TNFα and salubrinal.**
(TIFF)
