# Supplementary figures and images for: Distinctive Subcellular Inhibition of Cytokine-Induced Src by Salubrinal and Fluid Flow
Source: PLoS One. 2014 Aug 26;9(8):e105699. doi: 10.1371/journal.pone.0105699 (PMC4144888; doi:10.1371/journal.pone.0105699)

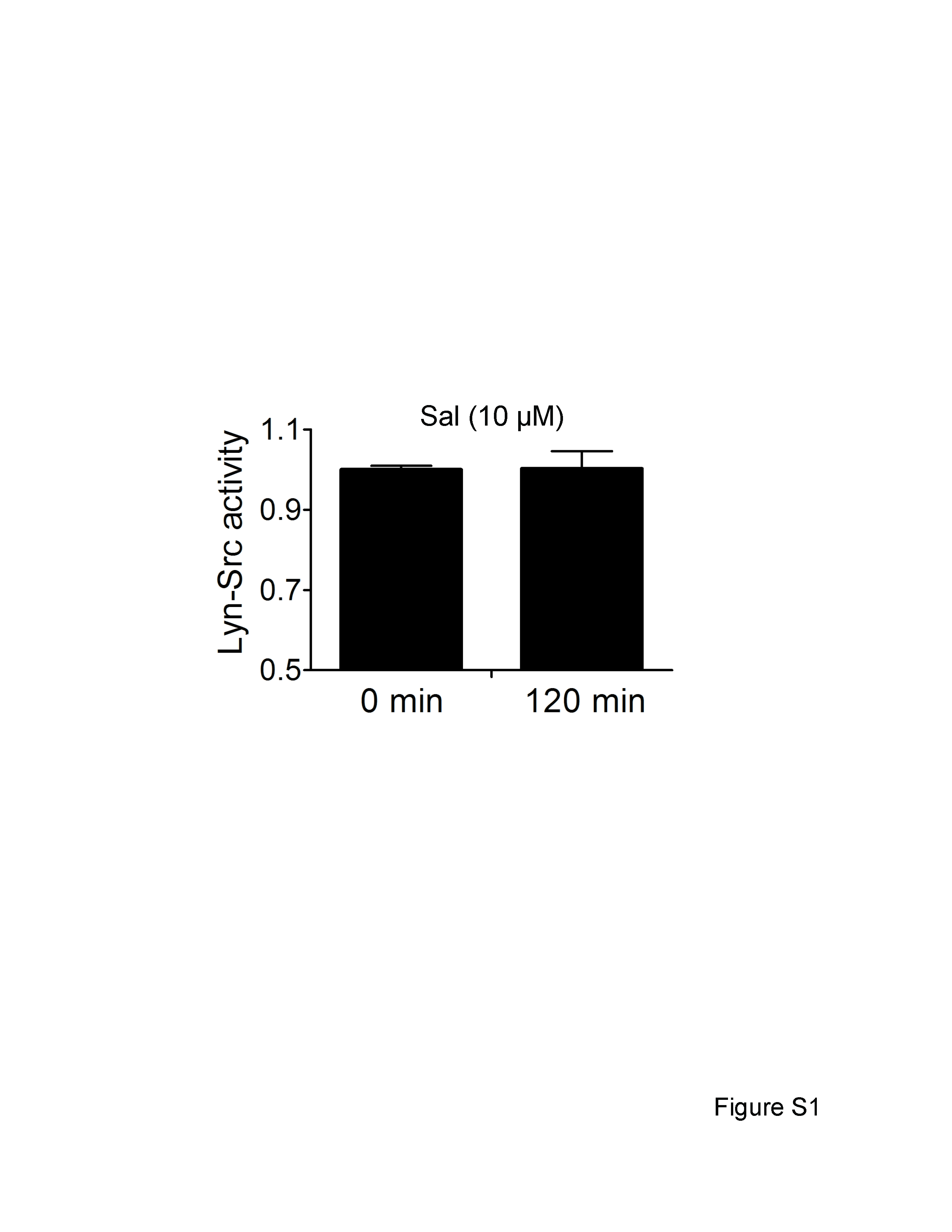

Supplement: Figure S1 — Lyn-Src response to salubrinal (10 µM). Two hour-imaging data shows that salubrinal does not affect Lyn-Src activity. n = 7 cells. (TIFF) [file pone.0105699.s001.tiff]

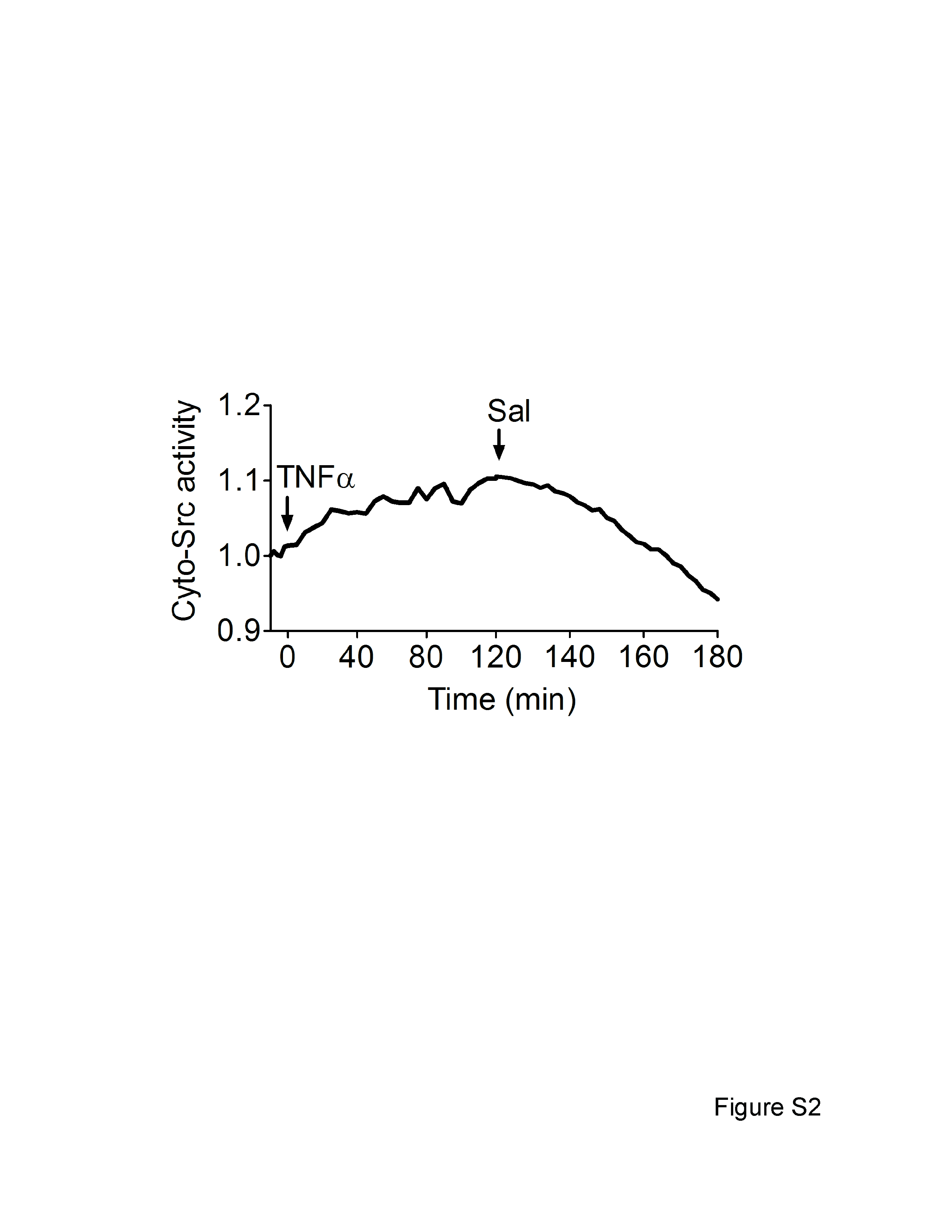

Supplement: Figure S2 — Cyto-Src activity of a representative cell in response to to TNFα and salubrinal. (TIFF) [file pone.0105699.s002.tiff]
